# Nitrogen addition strengthens the stabilizing effect of biodiversity on productivity by increasing plant trait diversity and species asynchrony in the artificial grassland communities

**DOI:** 10.3389/fpls.2023.1301461

**Published:** 2023-11-20

**Authors:** Ji Suonan, Xuwei Lu, Xiaona Li, Yann Hautier, Chao Wang

**Affiliations:** ^1^ College of Life Sciences, Qinghai Normal University, Xining, China; ^2^ Institute of Grassland, Flowers and Ecology, Beijing Academy of Agriculture and Forestry Sciences, Beijing, China; ^3^ Ecology and Biodiversity Group, Department of Biology, Utrecht University, Utrecht, Netherlands

**Keywords:** community stability, fast–slow traits, functional diversity, nitrogen addition, species stability, species asynchrony

## Abstract

**Background and aims:**

Nitrogen (N) enrichment usually weakens the stabilizing effect of biodiversity on productivity. However, previous studies focused on plant species richness and thus largely ignored the potential contributions of plant functional traits to stability, even though evidence is increasing that functional traits are stronger predictors than species richness of ecosystem functions.

**Methods:**

We conducted a common garden experiment manipulating plant species richness and N addition levels to quantify effects of N addition on relations between species richness and functional trait identity and diversity underpinning the ‘fast–slow’ economics spectrum and community stability.

**Results:**

Nitrogen addition had a minor effect on community stability but increased the positive effects of species richness on community stability. Increasing community stability was found in the species-rich communities dominated by fast species due to substantially increasing temporal mean productivity relative to its standard deviation. Furthermore, enhancement in ‘fast–slow’ functional diversity in species-rich communities dominated by fast species under N addition increased species asynchrony, resulting in a robust biodiversity–stability relationship under N addition the artificial grassland communities.

**Conclusion:**

The findings demonstrate mechanistic links between plant species richness, ‘fast–slow’ functional traits, and community stability under N addition, suggesting that dynamics of biodiversity–stability relations under global changes are the results of species-specific responses of ‘fast–slow’ traits on the plant economics spectrum.

## 1 Introduction

Nitrogen (N) deposition has increased considerably because of increases in anthropogenic activities, including increased combustion of fossil fuels and direct agricultural inputs (Galloway et al., 2008; Liu et al., 2013; Penuelas et al., 2013). The resulting eutrophication can weaken the stabilizing effect of biodiversity on primary productivity (Song and Yu, 2015; Zhang et al., 2016; Liu et al., 2019; Hautier et al., 2020). Stability of primary productivity is usually calculated as the ratio of mean productivity to its temporal standard deviation (Hautier et al., 2015; Hautier et al., 2020; Yan et al., 2021). The stabilizing effect of biodiversity is the result of two nonexclusive mechanisms: high species stability, characterized by stability of each species, and high species asynchrony, characterized by variations in interspecific responses to environmental fluctuations, with high species richness (Loreau and de Mazancourt, 2008; Thibaut and Connolly, 2013; Hautier et al., 2014).

Although most studies focus on plant species richness as a measure of plant diversity, plant functional trait identity and diversity along the ‘fast–slow’ economics spectrum are also important (Craven et al., 2018; Polley et al., 2020; Schnabel et al., 2021). Variation in leaf traits occurs along a single plant economics axis that is divided into acquisitive species with relatively fast growth and resource uptake (hereafter ‘fast’ species) and conservative species with relatively slow growth and resource uptake (hereafter ‘slow’ species) (Wright et al., 2004; Reich and Cornelissen, 2014). Fast species with high specific leaf area, leaf N concentration, and nitrogen/phosphorus (N/P) ratio and low leaf dry matter content could enhance community stability by increasing community temporal mean productivity, whereas slow species have opposite characteristics could enhance community stability by increasing community persistence (Reich and Cornelissen, 2014; Díaz et al., 2016; van der Plas et al., 2020). Plant functional traits stabilize community productivity because growth-related ‘fast–slow’ functional traits strongly influence plant productivity and persistence in grasslands (Diaz and Cabido, 2001; de Bello et al., 2010). Therefore, ecosystem functions may depend more on ‘fast–slow’ functional traits of species than on plant species richness (Hooper et al., 2005; Garnier et al., 2016; Polley et al., 2020).

Previous studies assessing N addition effects on community stability in grassland focused on plant species richness. In those studies, nitrogen addition usually reduces community stability by decreasing species richness-mediated species asynchrony and species stability (Hautier et al., 2014; Zhang et al., 2016; Hautier et al., 2020; Jia et al., 2022). However, previous studies ignored potential contributions of plant functional traits to community stability (de Bello et al., 2021). There are multiple mechanisms by which functional traits can affect community stability. First, the species-rich communities dominated by species with greater acquisitive trait can maintain higher community productivity than those dominated by species with higher conservative traits (Sun et al., 2006; Grigulis et al., 2013; Li et al., 2020), which can result in increasing community stability because of increased temporal mean productivity. However, the species-rich communities dominated by species with high slow trait values (i.e., high leaf dry matter content) are generally more stable over time (Lepš et al., 1982; Grime, 1998; Májeková et al., 2014; Craven et al., 2018), with relatively high community stability resulting from decreased variation of productivity from year to year. Moreover, high ‘fast-slow’ functional diversity (fast-slow FD), characterized by interspecific differences in resource utilization, can be correlated with high community stability because of overyielding (Hector et al., 2010; Yan et al., 2021). Similarly, high fast-slow FD also indicates that species can fluctuate independently from one another in fluctuating environments in the species-rich communities (i.e., relatively high species asynchrony) (Vergnon et al., 2009; Rocha et al., 2011; Adler et al., 2013; Lepš et al., 2018).

Communities composed of species with different fast–slow traits respond divergently to N addition (Pennings et al., 2005; Suding et al., 2005; Avolio et al., 2014). The mechanisms by which N addition affects community stability can be explained by differences in functional traits among plant species. Plants with higher acquisitive trait values responded sensitively to fluctuating environments (Craven et al., 2018), and plants in communities may shift from species with slow leaf economics to species with fast traits under N addition (Zhou et al., 2018; Zhang et al., 2019; Zheng et al., 2022), resulting in decreased species stability. In addition, the effects of N addition on community stability are correlated with either increased or decreased species asynchrony depending on whether N addition increased or decreased fast–slow FD in species-rich communities (Bongers et al., 2021). Nitrogen addition can decrease fast–slow FD by reducing species richness, which leads to a decline in species asynchrony (Hautier et al., 2020). However, nitrogen addition can also increase fast–slow FD because of divergent responses of different fast–slow functional traits when N addition does not change species richness (Palmroth et al., 2014), which leads to an increase in species asynchrony.

In this study, we used a common garden experiment to examine the effects of N addition on relations between plant species richness, ‘fast–slow’ functional trait identity and diversity, and community stability of productivity in assembled grassland communities. The common garden experiment manipulated plant species richness at five levels (1, 2, 4, 6, and 8 species) and N addition at two levels (0 and 6 g N m^−2^ year^−1^). The experiment was designed to answer the following two questions: (1) Does N addition affect relations between plant species richness, functional traits (‘fast–slow’ trait identity and diversity), and community stability (species stability and species asynchrony)? (2) What are the mechanisms by which N addition affects those relations (see **Table 1**)?

**Table 1:** Underlying mechanisms for relations between plant species richness, functional traits, and community stability.

Pathway	Hypotheses and mechanisms	References
**Fast–slow trait identity→species stability**	1. Species with high fast trait values increase species stability by increasing productivity of individual species over time; 2. Species with high slow trait values increase the stability of species productivity by decreasing variation in the productivity of individual species over time.	Sun et al., 2006; Májeková et al., 2014; Craven et al., 2018
**Fast–slow trait diversity→species asynchrony**	The possibility for asynchronous responses to fluctuating environments increases when the diversity of fast–slow traits is high in species-rich communities.	Lepš et al., 2018; Polley et al., 2020
**Species stability→community stability**	High species stability increases community stability because of high fast trait-induced high productivity or high slow trait-induced low variation in productivity over time.	Craven et al., 2018
**Species asynchrony→community stability**	High species asynchrony in response to fluctuating environments increases community stability because decreases in fast–slow trait values of some species are compensated for by increases in those of others, thus buffering temporal variations in the community-wide metric of fast–slow traits.	Craven et al., 2018

## 2 Materials and methods

### 2.1 Study site and experimental design

The study site was the National Grasses Variety Regional Experimental Station (40°10’45’’N, 116°26’13’’E, 50 m above sea level) on Xiaotang Mountain in Beijing, China. Mean annual temperature and precipitation is 11.8°C and 526 mm (2000–2018), respectively. In 2019, a common garden experiment was established within an 8 m × 12 m area in a split-plot experiment design with N addition and plant species richness as the main treatment factors. The experiment was composed of the following: two N levels (0 and 6 g N m^−2^ year^−1^) × four replicate blocks × 5 species richness levels per N treatment/replicate (four combinations per level of diversity with one, two, four, and six species and one with eight species, in total 17 combinations) = 136 pots (**Figure S1**). Each pot (30-cm diameter, 50-cm height) was filled with uniformly mixed soil (natural native grassland soil with stones and roots removed by sieving) and sand (3:1 soil: sand ratio), three holes (3-cm diameter) were set in the bottom to allow the pots to drain freely, and then buried in the ground without any shelter.

Eight plant species were used to build the artificial grassland communities (Wang et al., 2023). Seeds for each species were purchased commercially and germination rates were determined in a greenhouse (**Figure S1A**). To maintain the consistency of plant communities at the beginning of the experiment, sowing rate was determined based on the germination rate of each species in order to manipulate the species to have the same original proportion in the species-rich communities (**Figure S1A**). In each block, well-mixed seeds of the different combinations at different levels of species richness were distributed randomly among the 17 pots under ambient condition and N addition treatment, respectively. Community abundance was maintained at approximately 60 individuals in each pot by manual removal based on native grassland community abundance. Plants in pots were not re-sown after 2019 because the species were perennial. Weeds in pots were removed by hand once a month. Nitrogen was added as urea (12.86 g m^−2^ year^-1^, twice the amount of N deposition in Beijing) dissolved in water and applied by spraying on 1 May each year; the ambient conditions received an equal amount of N-free water.

### 2.2 Productivity and ‘fast–slow’ functional trait measurements

First, the abundance of each plant species in each pot was measured in 2019–2021. Second, plant height and leaf traits of three healthy individuals of each plant species in each pot were measured. Leaf traits of two leaves on individual plants were measured once at the peak of the growing season. Leaf traits were measured using standard methods according to Pérez-Harguindeguy et al. (2013). Spread leaves were scanned, and leaf mean width (LW) and area (LA) were determined using a Li−Cor 310 (Li−Cor Inc. USA). Leaves were weighed fresh, dried for 48 h at 65°C, and then weighed again to calculate leaf dry matter content (LDMC, g dry mass g^−1^ fresh mass). Specific leaf area (SLA, cm^2^ dry mass g^−1^) was calculated as the ratio of leaf area to its dry mass. Third, oven-dried leaf samples were ground, and leaf carbon (LCC, %) and N (LNC, %) concentration were determined with an elemental analyzer (Vario MACRO Cube. Germany). Leaf phosphorus concentration (LPC, %) was determined by inductively coupled plasma atomic emission spectroscopy (AutoAnalyzer 3. Germany). Finally, the leaf C/N, C/P, and N/P ratios were calculated. All the traits were measured in 2019 and 2020.

To measure community and individual productivity, the aboveground part of each pot was clipped in early September (peak of the growing season) from 2019 to 2021. All plants were clipped in each pot, sorted by species, oven−dried at 65°C to constant weight, and then community productivity was calculated. Average productivity from 2019 to 2021 was defined as temporal mean productivity. Because plants were not re-sown after the experiment began, clipping might have affected aboveground biomass in the following year and thus affected community stability (He et al., 2021; Wang et al., 2021). Fortunately, the plants selected in our experiment were all perennial species, aboveground biomass was clipped in the same way in all pots, and plant species richness did not change significantly across the three years (**Figure S2**). Hence, calculating community stability and its abiotic factors based on community productivity was reliable in our analysis.

### 2.3 Quantifying functional trait identity and diversity

Eight traits associated with the ‘fast-slow’ continuum of plant resource captures and usage (Lavorel and Grigulis, 2012; Grigulis et al., 2013) were selected to calculate the community-weighted mean (CWM) for each pot using the mean abundance-weighted values for each species. Then, the trait values were converted to standardized units (Z-scores). First, principal component analysis (PCA) was used to distinguish between fast and slow traits based on community-weighted mean traits using the “*vegan*” package in R software (**Figure S3**). The first PCA axis of CWM of functional traits (CWM fast-slow) captured 57.0% of the variation in the 8 traits and represented the fast–slow plant economics spectrum of communities, from those dominated by fast species with high plant height, SLA, leaf N, and N/P ratio and low LDMC and LA to those dominated by slow species with low plant height, SLA, leaf N, and N/P ratio and high LDMC and LA (**Figure S3**). Principal component analysis was also used to separate plant species into those with fast or with slow growth based on species-specific traits (**Figure S4**), it separated plant species into two slow species (*Chrysanthemum maximum* and *Allium schoenoprasum*) and six fast species (*Poa annua*, *Carex breviculmis*, *Medicago sativa*, *Astragalus adsurgens*, *Penstemon campanulatus*, *Dianthus barbatus*) based on relations with ‘fast–slow’ traits (**Figures S1**, **S4**), which indicated that the assembled grassland communities were fast growth and sensitive to N addition. Last, fast-slow FD was calculated as mean abundance-weighted distance of species to represent the range and distribution of traits in multidimensional niche space using the ‘*FD*’ package (Laliberte and Legendre, 2010).

### 2.4 Quantifying stability

Community stability of productivity was quantified as the ratio of community temporal mean productivity (μ*_T_*) to its standard deviation (σ*_T_*) in each pot over three years (2019–2021) as follows: *S_com_= μ_T_/σ_T_.* The ratio was widely used in many other studies (Hautier et al., 2014; Ma et al., 2017). According to recent theory, community stability can be mathematically partitioned into species stability and species asynchrony (Wang et al., 2019). Species stability (*S_sp_*) was calculated as the reciprocal of mean species variability (Wang et al., 2019): $S_{sp}=\frac{\mu_{T}}{\sum_{i}^{S}\sigma_{i}}$ , where σ*_i_* represents the temporal standard deviation of species *i*. Species asynchrony ( 1−∅ ) was defined as the reciprocal of a community-wide metric of species synchrony (Wang et al., 2019): $1-\emptyset =\frac{\sum_{i}^{S}\sigma_{i}}{\sigma_{T}}$ .

### 2.5 Statistical analyses

Linear mixed-effects models using the “*lme*” function (package “*nlme*”) (Pinheiro et al., 2007) were applied to test the effects of N addition and plant species richness on community productivity, CWM fast-slow, and fast-slow CWM. Nitrogen addition and plant species richness were fixed effects, and year, block, and plant combinations were random effects. Linear mixed-effects models were also used to examine the effects of N addition and plant species richness on community stability, species stability, and species asynchrony. Nitrogen addition and plant species richness were set as fixed effects, and block and plant combinations were set as random effects. Linear mixed-effects models were also used to test the relationship between community stability and abiotic factors. Nitrogen addition were set as fixed effects, and block and plant combinations were set as random effects. In order to test the interaction of N addition and plant species richness, analysis of covariance (ANCOVA) was used to test the differences in slopes of linear regressions between the ambient conditions and N addition treatments in the ‘*car*’ package in R software (Fox and Weisberg, 2019). Moreover, we compared the plant functional traits between the ambient conditions and N addition treatment in different species by paired-samples t test.

A multi-group structural equation model (SEM_m_) was used to examine direct and indirect relations between plant species richness, CWM fast-slow and fast-slow FD (temporal mean across two years), and community stability under different levels of N addition. The ‘*piecewise-SEM*’ package in R software was used to construct the SEM_m_ (Lefcheck, 2016). The SEM_m_ was also constructed to determine whether the stabilizing effects on productivity operated via the two components of the invariability measure, temporal mean and standard deviation of productivity under ambient condition and N addition treatment. In the second model, direct and indirect paths from species richness, CWM fast–slow, fast–slow FD, and species asynchrony were added to the temporal mean and standard deviation of productivity. The models were assessed by *Fisher’s C* statistic, Akaike information criterion (*AIC*), *p* value, and degrees of freedom (*df*) values. Last, models with the lowest Fisher’s *C* and *AIC* values and higher *p* value (*p*≥0.05) were selected. In addition, pathways showed one arrow in the final SEM_m_ when the relationship showed no significant difference between the ambient condition and N addition treatment, but it divided into two arrows when the relationship under N addition treatment significantly different from that in ambient condition.

All statistical analyses and graph preparations were performed using R 3.2.2 (R Core Team, 2018). Effects were considered significant at *p* ≤ 0.05 or marginally significant at 0.05< *p* ≤ 0.10.

## 3 Results

### 3.1 Productivity and stability response to nitrogen addition and plant species richness

**Figure 1:**
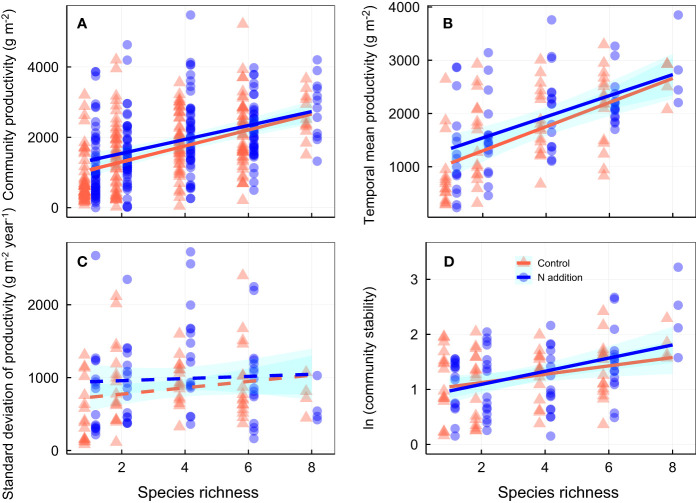
Effects of nitrogen addition and species richness on **(A)** community productivity (productivity in each pot each year), **(B)** temporal mean productivity (average productivity over three years in each pot), **(C)** standard deviation of temporal mean productivity, and **(D)** community stability. Control, orange points and lines; nitrogen addition, blue points and lines. Solid lines, significant relations (*p* ≤ 0.05); dashed lines, nonsignificant relations (*p* > 0.05). Shaded areas indicate 95% confidence intervals of the regression lines. Parameters for analysis of covariance and linear regression are shown in **Tables S2**, **S3**, respectively.

**Figure 2:**
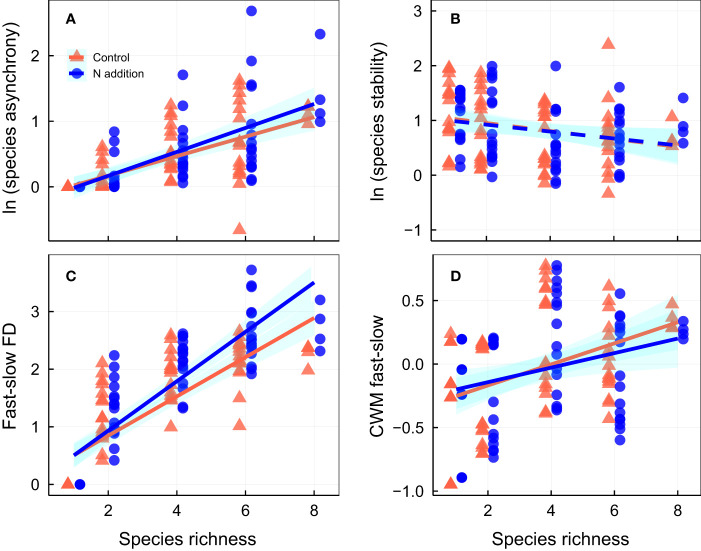
Effects of nitrogen addition and species richness on **(A)** species asynchrony, **(B)** species stability, **(C)** fast-slow functional diversity (fast-slow FD), and **(D)** community-weighted mean of fast-slow traits (CWM fast-slow). CWM fast-slow in the figures for each community the extracted first principal component from PCA. Control, orange points and lines; nitrogen addition, blue points and lines. Solid lines, significant relations (*p* ≤ 0.05); dashed lines, nonsignificant relations (*p* > 0.05). Shaded areas indicate 95% confidence intervals of the regression lines. Parameters for the analysis of covariance and linear regression are shown in **Tables S2**, **S4**, respectively.

Over the 3-year experimental period, nitrogen addition significantly increased community productivity, and species richness was positively associated with temporal mean community productivity, although the effects were largely independent of one another (**Figures 1A, B**; **Tables S1**, **S2**). Neither N addition nor species richness significantly affected standard deviation of productivity (**Figure 1C**; **Table S1**). Although N addition had minor effects on community stability (**Table S1**), species richness was positively associated with community stability (**Figure 1D**; **Table S4**). Nitrogen addition strengthened the relation between species richness and community stability and species asynchrony, resulting in a significant N addition × species richness interaction term (**Figures 1D**, **2A**; **Tables S1**, **S2**). In addition, nitrogen addition and species richness did not affect species stability (**Figures 2A, B**; **Table S1**). However, nitrogen addition marginally strengthened the relation between species richness and species asynchrony, resulting in a significant N addition × species richness interaction term (**Figure 2A**; **Tables S1**, **S2**), indicating the positive effect of species richness on species asynchrony enhanced under N addition in the species-rich communities.

### 3.2 Functional traits response to nitrogen addition and plant species richness

Nitrogen addition had a minor effect on fast–slow FD, but species richness was positively associated with fast–slow FD (**Figure 2C**; **Tables S1**, **S5**). Nitrogen addition strengthened the relation between species richness and fast–slow FD, resulting in a significant N addition × species richness interaction term (**Figure 2C**; **Tables S1**, **S2**), indicating the positive effect of species richness on fast–slow FD increased under N addition in species-rich communities. Nitrogen addition did not affect the CWM fast–slow (**Figure 2D**; **Table S1**), species richness was positively associated with CWM fast–slow (**Figure 2D**; **Tables S1**, **S5**), but the effects of N addition were not interacted with plant species richness (**Figure 2D**; **Table S1**). Moreover, nitrogen addition did not affect most CWM trait values, but CWM of LA and LW increased and CWM of SLA decreased with N addition (**Table S3**). Species richness was positively associated with most CWM trait values (**Table S3**).

### 3.3 Ecological factors influencing community stability

**Figure 3:**
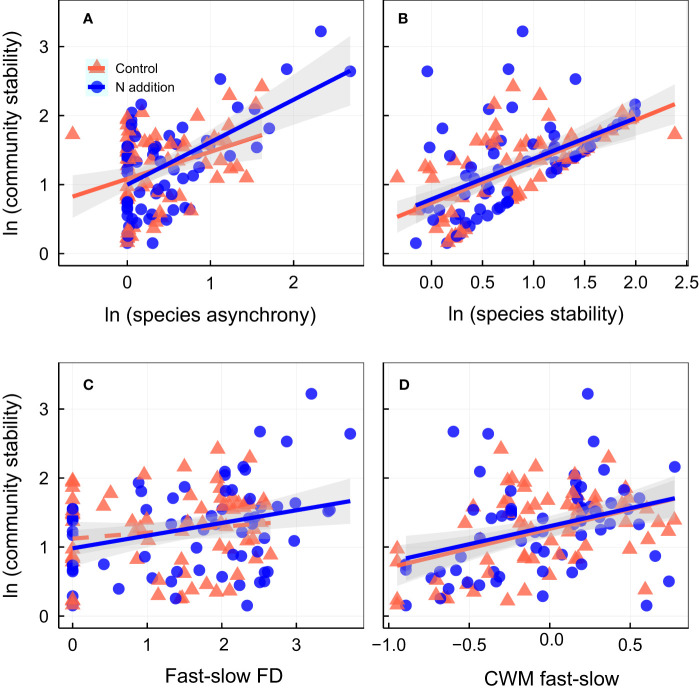
Relationships between community stability and **(A)** species asynchrony, **(B)** species stability, **(C)** fast-slow functional diversity (fast-slow FD), and **(D)** community-weighted mean of fast-slow traits (CWM fast-slow). Control, orange points and lines; nitrogen addition, blue points and lines. Solid lines, significant relations (*p* ≤ 0.05); dashed lines, nonsignificant relations (*p* > 0.05). Shaded areas indicate 95% confidence intervals of the regression lines. Parameters for the analysis of covariance and linear regression are shown in **Tables S2**, **S6**, respectively.

**Figure 4:**
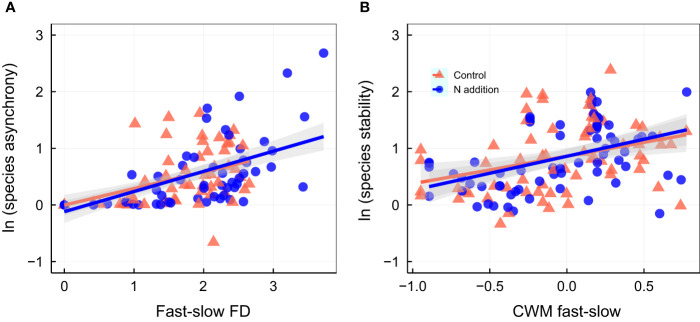
Relationship between species asynchrony and fast-slow functional diversity [fast-slow FD, **(A)**]; relationship between species stability and community-weighted mean of fast-slow traits [(CWM fast-slow, **(B)**]. Control, orange points and lines; nitrogen addition, blue points and lines. Solid lines, significant relations (*p* ≤ 0.05); dashed lines, nonsignificant relations (*p* > 0.05). Shaded areas indicate 95% confidence intervals of the regression lines. Parameters for the analysis of covariance and linear regression are shown in **Tables S2**, **S7**, respectively.

Community stability was positively associated with species stability and CWM fast–slow (higher CWM fast–slow represents greater acquisitive trait) under ambient condition (**Figures 3B, D**; **Table S6**). Under N addition, community stability was positively associated with species asynchrony (**Figure 3A**), species stability (**Figure 3B**), fast–slow FD (**Figure 3C**), and CWM fast–slow (**Figure 3D**; **Table S6**). Species asynchrony was positively associated with the fast–slow FD under N addition (**Figure 4A**; **Table S7**), and species stability was positively associated with the CWM fast–slow traits in both control and N addition (**Figure 4B**; **Table S7**). In addition, temporal mean productivity was positively associated with the CWM fast–slow and fast–slow FD in both ambient condition and N addition treatment (**Figures S4A, C**). It was positively associated with CWM of plant height, SLA, and leaf N content but negatively associated with CWM of LDMC (**Figure S5**). Moreover, the standard deviation of productivity exhibited no significant relationships with the CWM of plant height, LA, LDMC, SLA, or leaf N content or the N/P ratio (**Figure S6**).

**Figure 5:**
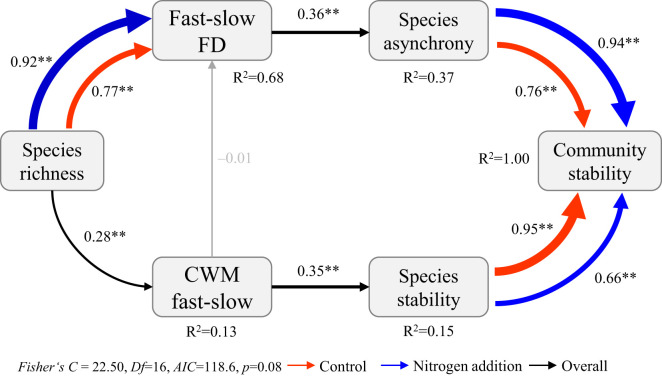
Multi-group structural equation model exploring the effects of species richness, the community-weighted mean (CWM fast-slow) and the diversity (fast-slow FD) of fast-slow traits on species stability, species asynchrony, and community stability under different levels of nitrogen addition. Boxes represent measured variables, and arrows represent relations between variables. Solid and dashed arrows represent positive and negative pathways, respectively. Black, red, and blue arrows represent significant (*p* ≤ 0.05) pathways overall, and under ambient condition and nitrogen addition treatments, respectively. Grey arrows represent nonsignificant pathways (*p* > 0.05). Standardized path coefficients are given next to each path. *0.01<*p*≤0.05, **p≤0.01.

**Figure 6:**
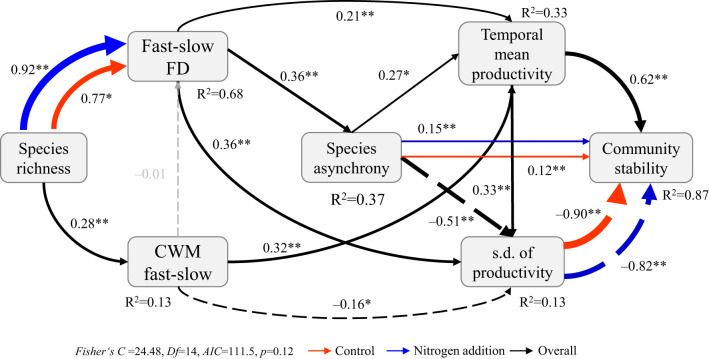
Multi-group structural equation model exploring the effects of species richness, community-weighted mean of fast–slow traits (CWM fast–slow), and fast–slow functional diversity (fast–slow FD) on species asynchrony, temporal mean and variation (s.d. of productivity) in productivity, and community stability under different levels of nitrogen addition. Boxes represent measured variables, and arrows represent relations between variables. Solid and dashed arrows represent positive and negative pathways, respectively. Black, red, and blue arrows represent significant (*p* ≤ 0.05) pathways overall and in control and nitrogen addition treatments, respectively. Grey arrows represent nonsignificant pathways (*p* > 0.05). Standardized path coefficients are given next to each path. *0.01<*p*≤0.05, **p≤0.01.

The SEM_m_ indicated that community stability–species stability, community stability–species asynchrony, and fast–slow FD–species richness relations were significantly affected by N addition (**Figures 5**, **6**; **Table S9**). Specifically, both species stability and species asynchrony were key factors mediating the relation between species richness and community stability, with species stability driven by the CWM fast–slow and species asynchrony driven by fast–slow FD under N addition (**Figure 5**). Moreover, species richness increased temporal mean productivity by increasing the CWM fast–slow and decreased productivity variability, which resulted in a positive relation between biodiversity and stability under ambient condition (**Figure 6**; **Table S9**). However, species asynchrony–community stability, standard deviation of temporal mean productivity–community stability, and species richness–fast-slow FD relations were significantly affected by N addition (**Figure S7B**; **Table S9**). Species richness reduced the standard deviation of temporal mean productivity by increasing fast–slow FD, which resulted in a strengthened biodiversity–stability relation under N addition (**Figure 6**).

## 4 Discussion

The intrinsic links among species richness, functional traits (i.e., CWM fast–slow and fast–slow FD), and community stability (i.e., species stability and species asynchrony) were identified, and mechanisms underlying effects of N addition on the biodiversity–stability relation were clarified. In particular, species richness increased community stability mainly by increasing the CWM fast–slow under ambient condition. Although N addition did not significantly affect fast–slow FD and community stability, there was a positive interaction with species richness. Importantly, nitrogen addition strengthened the relation between species richness and community stability by increasing fast–slow FD and species asynchrony in the species-rich communities dominated by fast species. The results provide a novel perspective to understand potential mechanisms linking functional traits and community stability under global change.

### 4.1 Biodiversity increased community stability by increasing fast–slow trait identity

Species richness increased community stability by increasing species stability under ambient condition (**Figure 5**). The result is similar to theoretical predictions and experimental investigations that show a weak relation between biodiversity and stability when community stability is driven by dominant species (Tilman, 1996; Grman et al., 2010; Yang et al., 2012; Xu et al., 2015; Wang Y. et al., 2020). In addition, species stability was positively associated with CWM of plant height, leaf N content, and leaf N/P ratio (**Figure S9**). Thus, the species-rich communities dominated by species with relatively high fast trait values maintained relatively high species stability. Our results showed that species richness increased species stability by increasing the CWM fast–slow (**Figure 5**). The result contrasts with previous studies who showed that communities dominated by slow species exist stronger resistance response to fluctuating environments (Grime, 1998; Májeková et al., 2014; Craven et al., 2018). In further analysis, CWM of plant height, SLA, and leaf N content were positively associated but LDMC was negatively associated with temporal mean productivity rather than its standard deviation over time (**Figures S5**, **S6**). Therefore, the species-rich communities dominated by species with high acquisitive traits increased community stability by increasing temporal mean productivity (**Figure S9**). As with the effects of species richness on temporal mean productivity over time, those effects might be explained by selection effects in the species-rich communities (Loreau and Hector, 2001), and thus, the positive association between community stability and CWM fast–slow might be because more fast species were selected in the species-rich communities.

### 4.2 Nitrogen addition strengthened the biodiversity−stability relations

One key finding in our study was that the interaction between N addition and species richness positively affected community stability, with N addition increasing community stability in the species-rich communities dominated by fast species (**Figures 1D**, **5**). Nitrogen addition strengthened the biodiversity−stability relation via a combination of two processes (**Figure 5**; **Table S8**). First, positive relations between species stability and community stability were not significantly affected by N addition. Second, the weak relation between community stability and species asynchrony under ambient condition became a significant positive relation after N addition. The results are not consistent with those of Hautier et al. (2014); Hautier et al. (2020) who found that N addition weakened the contribution of species richness to community stability. Hautier et al. (2020) proposed that a relatively weak positive relation between species richness and species asynchrony was the result of decreasing species richness under N addition; whereas a relatively strong negative relation between species richness and species stability under N addition may be caused by shifts in functional traits in communities from species with conservative traits to those with acquisition traits (Májeková et al., 2014; Isbell et al., 2015). There are two primary differences between our experiment and previous studies. First, the assembled communities in our experiment were dominated by fast growth species, nitrogen addition diversified species acquisitive traits and increased functional diversity in species-rich communities, which led to greater asynchronous dynamics to fluctuating environments and lower productivity variation over time in species-rich communities, resulting in a stronger relation between community stability and species asynchrony under N addition. Second, nitrogen addition did not significantly affect species richness and CWM fast–slow in our study (**Table S1**), resulting in no shifts in functional traits in species-rich communities, and thus and not changes in the relation between species richness and species stability (**Figure 2A**).

Asynchrony between species is generally attributed to divergent responses of species with different functional traits to environmental fluctuations (Ives et al., 1999; Loreau and de Mazancourt, 2008; Loreau and de Mazancourt, 2013). Hence, species asynchrony could have increased because of divergent responses of species-specific fast–slow traits to N addition in our common garden experiment (**Figure S10**; **Table S10**) (Adler et al., 2013; Lepš et al., 2018). It has been proved that species with various fast–slow traits can stabilize the productivity of mixed-species communities by divergent responses to N addition (Pennings et al., 2005; Suding et al., 2005; Avolio et al., 2014). For example, the legumes (*Medicago sativa* and *Astragalus adsurgens*) in the species-rich communities can perform symbiotic N fixation (Bordeleau and Prévost, 1994). Thus, because growth is not limited by N, legume fast–slow traits might not be influenced by N addition (**Figure S10**). However, most grassland ecosystems are limited by N (Elser et al., 2007; Harpole et al., 2011; Fay et al., 2015), and growth of most species in grasslands (i.e., grasses and forbs) is closely associated with soil available nutrients (Ren et al., 2016). To maximize nutrient use for plant growth (Reich and Cornelissen, 2014), acquisitive trait values (i.e., higher specific leaf area) of grasses, sedges, and forbs in the species-rich communities enhanced under N addition (**Figure S10**), possibly because of increases in soil available N and P (LeBauer and Treseder, 2008; Wang C. et al., 2020; Radujković et al., 2021). Thus, the divergent responses of functional traits under N addition led to the increase in fast–slow FD in the species-rich communities.

Moreover, higher fast-slow FD in the species-rich communities under N addition indicated that an increase in dissimilarity in functional traits among plant species, higher differentiation in functional traits in species-rich communities exhibited greater asynchronous dynamics in fluctuating environments (Craven et al., 2018; Polley et al., 2020; Schnabel et al., 2021). Hence, greater species asynchrony stabilized productivity because of its stronger negative effects on the standard deviation of productivity over time than its positive effects on temporal mean productivity under N addition (**Figure 6**; **Table S9**). Overall, the strengthened biodiversity–stability relation under N addition was caused by reduction in the standard deviation of productivity in fluctuating environments that was associated with the increase in fast-slow FD in the species-rich communities dominated by fast species.

### 4.3 Limitations

Furthermore, our study provides novel perspectives from which to understand potential links among biodiversity and community stability under N addition, but the conclusion in our common garden experiment differs from that in most previous studies was partly attributed to experimental duration, which could affect the response of community stability to N addition in two aspects. First, the artificial grassland communities were built through random combinations of different species, but some species in the mixed community were not native species (i.e., *Chrysanthemum maximum*). Hence, the artificial communities did not develop to s stable stage in 3 years, there were significant differences between the artificial and natural grasslands, which might have led to differences in results with previous studies. Hence, our results provide a new sight to investigate the relationship between plant functional traits and community temporal stability, but simply applying our conclusions to natural grasslands requires caution. Second, dynamic of climate conditions (i.e., mean annual temperature and precipitation) during 3 period years may not cover the climate variation in this site, which could also lead to the results in our experiment different from the others. Fortunately, each treatment in our experiment was conducted under the same climate condition, and our analysis focused on the comparison in the effects of biodiversity under ambient condition and nitrogen addition along the plant species richness gradients, which lead our conclusion different from the conclusion from other grassland communities but convinced.

## 5 Conclusions

The common garden experiment was an important step forward in understanding the biodiversity–stability relation because it demonstrated mechanistic links between species richness, functional traits (i.e., functional trait identity and diversity), and community stability (i.e., species stability and species asynchrony) under different levels of N addition. Specifically, a positive relation was identified between species stability and fast–slow trait identity, highlighting that species richness increased community stability because of high temporal mean productivity in the species-rich communities dominated by fast species. In particular, nitrogen addition strengthened the biodiversity–stability relation by increasing fast–slow functional diversity and species asynchrony in the species-rich communities dominated by fast species in the artificial grassland communities. The links suggest that N addition may increase grassland ecosystem stability when it does not alter species richness, and thus, appropriate nutrient addition may be an effective method to restore the degraded grasslands by using legume species.

## Data availability statement

The raw data supporting the conclusions of this article will be made available by the authors, without undue reservation.

## Author contributions

JS: Data curation, Writing – review & editing, Investigation, Resources. XLu: Formal Analysis, Writing – original draft, Data curation, Investigation. XLi: Writing – review & editing. YH: Writing – review & editing. CW: Formal Analysis, Writing – original draft, Methodology, Visualization, Conceptualization.
