# Supplementary material for: Nitrogen addition strengthens the stabilizing effect of biodiversity on productivity by increasing plant trait diversity and species asynchrony in the artificial grassland communities
Source: Front Plant Sci. 2023 Nov 20;14:1301461. doi: 10.3389/fpls.2023.1301461 (PMC10694273; doi:10.3389/fpls.2023.1301461)

**Supplementary information**

**TABLE S1** Results for the effect of nitrogen addition and plant diversity on community productivity, stability, and functional traits. Bold fonts represent significant effects.

| Attributes | denDF | *F* value | | | *p* value | | |
| --- | --- | --- | --- | --- | --- | --- | --- |
|  |  | **Nitrogen** | **Diversity** | **Nitrogen*Diversity** | **Nitrogen** | **Diversity** | **Nitrogen*Diversity** |
| Community productivity | 351 | 4.94 | 107.6 | 0.48 | **0.03** | **<0.01** | 0.49 |
| Temporal mean productivity | 117 | 2.76 | 60.28 | 0.27 | 0.10 | **<0.01** | 0.61 |
| Standard deviation of temporal mean productivity | 117 | 2.12 | 1.70 | 0.42 | 0.16 | 0.22 | 0.19 |
| Community stability | 117 | 1.07 | 23.67 | 5.13 | 0.30 | **<0.01** | 0.01 |
| Species asynchrony | 85 | 1.55 | 46.26 | 3.06 | 0.22 | **<0.01** | 0.08 |
| Species stability | 85 | <0.01 | 1.86 | 0.10 | 0.98 | 0.18 | 0.75 |
| Fast-slow functional diversity | 85 | <0.01 | 57.17 | 4.02 | 0.99 | **<0.01** | **0.04** |
| Community weighted mean of fast-slow traits | 85 | 1.23 | 13.39 | 0.05 | 0.27 | **<0.01** | 0.83 |

**TABLE S2** Results for analysis of covariance in Figure 1-4. Bold fonts represent significant effects.

| Relationships | Treatment | DF | *F* value | *p* value |
| --- | --- | --- | --- | --- |
| Community productivity ←Species richness | Nitrogen addition | 1 | 4.64 | **0.03** |
|  | Species richness | 1 | 100.3 | **<0.01** |
|  | Nitrogen addition*Species richness | 1 | 0.45 | 0.51 |
| Temporal mean productivity ← Species richness | Nitrogen addition | 1 | 2.71 | **0.10** |
|  | Species richness | 1 | 58.67 | **<0.01** |
|  | Nitrogen addition*Species richness | 1 | 0.26 | 0.61 |
| Standard deviation of temporal mean productivity ←Species richness | Nitrogen addition | 1 | 2.12 | 0.15 |
|  | Species richness | 1 | 1.70 | 0.19 |
|  | Nitrogen addition*Species richness | 1 | 0.42 | 0.52 |
| Community stability ←Species richness | Nitrogen addition | 1 | 0.98 | 0.33 |
|  | Species richness | 1 | 22.3 | **<0.01** |
|  | Nitrogen addition*Species richness | 1 | 4.71 | **0.03** |
| Species asynchrony ←Species richness | Nitrogen addition | 1 | 1.48 | 0.67 |
|  | Species richness | 1 | 45.57 | **<0.01** |
|  | Nitrogen addition*Species richness | 1 | 2.92 | **0.08** |
| Species stability ←Species richness | Nitrogen addition | 1 | 0.12 | 0.73 |
|  | Species richness | 1 | 8.96 | **<0.01** |
|  | Nitrogen addition*Species richness | 1 | 0.02 | 0.88 |
| Fast-slow functional diversity ←Species richness | Nitrogen addition | 1 | 5.41 | **0.02** |
|  | Species richness | 1 | 306.2 | **<0.01** |
|  | Nitrogen addition*Species richness | 1 | 4.03 | **0.04** |
| Community weighted mean of fast-slow traits ←Species richness | Nitrogen addition | 1 | 0.04 | 0.85 |
|  | Species richness | 1 | 19.46 | **<0.01** |
|  | Nitrogen addition*Species richness | 1 | 0.65 | 0.41 |
| Community stability ←Species asynchrony | Nitrogen addition | 1 | 0.07 | 0.79 |
|  | Species asynchrony | 1 | 114.5 | **<0.01** |
|  | Nitrogen addition* Species asynchrony | 1 | 1.24 | 0.27 |
| Community stability ←Species stability | Nitrogen addition | 1 | 1.19 | 0.28 |
|  | Species stability | 1 | 19.27 | **<0.01** |
|  | Nitrogen addition*Species stability | 1 | 0.05 | 0.82 |
| Community stability ←Fast-slow functional diversity | Nitrogen addition | 1 | <0.01 | 0.98 |
|  | Fast-slow functional diversity | 1 | 8.19 | **<0.01** |
|  | Nitrogen addition* Fast-slow functional diversity | 1 | 0.91 | 0.35 |
| Community stability ←Community weighted mean of fast-slow traits | Nitrogen addition | 1 | 0.01 | 0.95 |
|  | Community weighted mean of fast-slow traits | 1 | 32.4 | **<0.01** |
|  | Nitrogen addition* Community weighted mean of fast-slow traits | 1 | 0.14 | 0.70 |
| Species asynchrony ←Fast-slow functional diversity | Nitrogen addition | 1 | 0.11 | 0.75 |
|  | Fast-slow functional diversity | 1 | 78.8 | **<0.01** |
|  | Nitrogen addition*Fast-slow functional diversity | 1 | 0.69 | 0.41 |
| Species stability ←Community weighted mean of fast-slow traits | Nitrogen addition | 1 | 0.01 | 0.94 |
|  | Species richness | 1 | 24.2 | **<0.01** |
|  | Nitrogen addition* Community weighted mean of fast-slow traits | 1 | 0.26 | 0.61 |

**TABLE S3** Results for the effect of nitrogen addition and plant diversity on functional traits at the community levels. Bold fonts represent significant effects.

| Functional traits | | denDF | *F* value | | | *p* value | | |
| --- | --- | --- | --- | --- | --- | --- | --- | --- |
|  |  |  | **Nitrogen** | **Diversity** | **Nitrogen*Diversity** | **Nitrogen** | **Diversity** | **Nitrogen*Diversity** |
|  | Plant height | 261 | 0.16 | 0.48 | <0.01 | 0.69 | 0.49 | 0.99 |
|  | Leaf area | 261 | 4.09 | 5.42 | 0.03 | **0.04** | **0.02** | 0.87 |
|  | Leaf dry matter content | 261 | 0.77 | 6.70 | 0.21 | 0.38 | **<0.01** | 0.65 |
|  | Specific leaf area | 261 | 4.19 | 3.48 | 0.59 | **0.04** | **0.06** | 0.44 |
|  | Leaf nitrogen content | 261 | 0.07 | 3.45 | 0.05 | 0.79 | **0.06** | 0.82 |
|  | Leaf nitrogen/phosphorus ratio | 261 | 0.07 | 4.88 | 0.04 | 0.79 | **0.02** | 0.83 |
|  | Leaf mean width | 261 | 3.56 | 0.01 | 0.24 | **0.06** | 0.92 | 0.62 |
|  | Leaf phosphorus content | 261 | 2.06 | 0.03 | 4.76 | 0.15 | 0.85 | **0.03** |

**TABLE S4** Results for linear regression in Figure 1. The R^2^(m) and R^2^(c) represent model variations explained by fixed effects and the combination of fixed and random effects, respectively. Asterisk represents that coefficients are significant: ***p* ≤ 0. 01, **p* ≤ 0.05.

| Relationships | Treatment | DF | R^2^(m) | R^2^(c) |
| --- | --- | --- | --- | --- |
| Community productivity ←Species richness | Control | 202 | 0.20** | 0.36** |
|  | Nitrogen addition | 202 | 0.16** | 0.28** |
| Temporal mean productivity ← Species richness | Control | 66 | 0.33** | 0.40** |
|  | Nitrogen addition | 66 | 0.27** | 0.27** |
| Standard deviation of temporal mean productivity ←Species richness | Control | 66 | 0.04 | <0.01 |
|  | Nitrogen addition | 66 | <0.01 | <0.01 |
| Community stability ←Species richness | Control | 66 | 0.09** | 0.41** |
|  | Nitrogen addition | 66 | 0.17** | 0.50** |

**TABLE S5** Results for linear regression in Figure 2. The R^2^(m) and R^2^(c) represent model variations explained by fixed effects and the combination of fixed and random effects, respectively. Asterisk represents that coefficients are significant: ***p* ≤ 0. 01, **p* ≤ 0.05.

| Relationships | Treatment | DF | R^2^(m) | R^2^(c) |
| --- | --- | --- | --- | --- |
| Species asynchrony ←Species richness | Control | 66 | 0.44** | 0.44** |
|  | Nitrogen addition | 66 | 0.45** | 0.45** |
| Species stability ← Species richness | Control | 66 | 0.07* | 0.07* |
|  | Nitrogen addition | 66 | 0.06* | 0.07* |
| Fast-slow functional diversity ←Species richness | Control | 66 | 0.66** | 0.66** |
|  | Nitrogen addition | 66 | 0.73** | 0.73** |
| Community weighted mean fast-slow traits ←Species richness | Control | 66 | 0.18** | 0.20** |
|  | Nitrogen addition | 66 | 0.09* | 0.10* |

**TABLE S6** Results for linear regression in Figure 3. The R^2^(m) and R^2^(c) represent model variations explained by fixed effects and the combination of fixed and random effects, respectively. Asterisk represents that coefficients are significant: ***p* ≤ 0. 01, **p* ≤ 0.05.

| Relationships | Treatment | DF | R^2^(m) | R^2^(c) |
| --- | --- | --- | --- | --- |
| Community stability ← Species asynchrony | Control | 66 | 0.12** | 0.12** |
|  | Nitrogen addition | 66 | 0.33** | 0.33** |
| Community stability ←Species stability | Control | 66 | 0.42** | 0.42** |
|  | Nitrogen addition | 66 | 0.28** | 0.28** |
| Community stability ← Fast-slow functional diversity | Control | 66 | 0.02 | 0.02 |
|  | Nitrogen addition | 66 | 0.09** | 0.09** |
| Community stability ← Community weighted mean fast-slow traits | Control | 66 | 0.18** | 0.19** |
|  | Nitrogen addition | 66 | 0.12** | 0.12** |

**TABLE S7** Results for linear regression in Figure 4. The R^2^(m) and R^2^(c) represent model variations explained by fixed effects and the combination of fixed and random effects, respectively. Asterisk represents that coefficients are significant: ***p* ≤ 0. 01, **p* ≤ 0.05.

| Relationships | Treatment | DF | R^2^(m) | R^2^(c) |
| --- | --- | --- | --- | --- |
| Species asynchrony ←Fast-slow functional diversity | Control | 66 | 0.30** | 0.30** |
|  | Nitrogen addition | 66 | 0.43** | 0.44** |
| Species stability ←Community weighted mean fast-slow traits | Control | 66 | 0.12** | 0.12** |
|  | Nitrogen addition | 66 | 0.20** | 0.21** |

**TABLE S8** Results for multi-group structural equation model of Figure 5. Treatment represent different N addition levels. CWM, community weighted mean; FD, functional diversity. Bold fonts represent significant effects.

| Response | Predictor | Test Stat | DF | *p* value |
| --- | --- | --- | --- | --- |
| Community stability | Species stability : Treatment | 580.0 | 1 | **0.04** |
| Community stability | Species asynchrony : Treatment | 580.0 | 1 | **<0.01** |
| Species stability | CWM fast-slow : Treatment | 7.3 | 1 | 0.61 |
| Species asynchrony | Fast-slow FD : Treatment | 14.9 | 1 | 0.41 |
| CWM fast-slow | Species richness : Treatment | 3.2 | 1 | 0.42 |
| Fast-slow FD | CWM fast-slow : Treatment | 83.9 | 1 | 0.59 |
| Fast-slow FD | Species richness : Treatment | 83.9 | 1 | **0.08** |

**TABLE S9** Results for multi-group structural equation model of Figure 6. Treatment represent different N addition levels. CWM, community weighted mean; FD, functional diversity; s.d., standard deviation. Bold fonts represent significant effects.

| Response | Predictor | Test Stat | DF | *p* value |
| --- | --- | --- | --- | --- |
| Community stability | Temporal mean productivity : Treatment | 16.7 | 1 | 0.71 |
| Community stability | s.d. of productivity : Treatment | 16.7 | 1 | **0.04** |
| Community stability | Species asynchrony : Treatment | 16.7 | 1 | **0.05** |
| Temporal mean productivity | Species asynchrony : Treatment | 207.7 | 1 | 0.85 |
| Temporal mean productivity | CWM fast-slow : Treatment | 207.7 | 1 | 0.25 |
| Temporal mean productivity | Fast-slow FD : Treatment | 207.7 | 1 | 0.73 |
| Species asynchrony | Fast-slow FD : Treatment | 14.9 | 1 | 0.41 |
| s.d. of productivity | Species asynchrony : Treatment | 16.0 | 1 | 0.25 |
| s.d. of productivity | CWM fast-slow : Treatment | 384.2 | 1 | 0.16 |
| s.d. of productivity | Fast-slow FD : Treatment | 384.2 | 1 | 0.93 |
| CWM fast-slow | Species richness : Treatment | 384.2 | 1 | 0.42 |
| Fast-slow FD | CWM fast-slow : Treatment | 83.9 | 1 | 0.59 |
| Fast-slow FD | Species richness : Treatment | 83.9 | 1 | **0.08** |

**TABLE S10** Results for the effect of nitrogen addition and plant species on functional traits at the species levels. Bold fonts represent significant effects.

| Functional traits | | denDF | *F* value | | | *p* value | | |
| --- | --- | --- | --- | --- | --- | --- | --- | --- |
|  |  |  | **Nitrogen** | **Species** | **Nitrogen* Species** | **Nitrogen** | **Species** | **Nitrogen* Species** |
|  | Plant height | 593 | 29.43 | 182.5 | 0.99 | **<0.01** | **<0.01** | 0.44 |
|  | Leaf area | 593 | 3.22 | 21.87 | 0.88 | **0.03** | **<0.01** | 0.52 |
|  | Leaf dry matter content | 593 | 2.65 | 66.24 | 0.20 | 0.10 | **<0.01** | 0.99 |
|  | Specific leaf area | 593 | 0.11 | 3.67 | 0.70 | 0.74 | **<0.01** | 0.68 |
|  | Leaf nitrogen content | 593 | 0.05 | 189.1 | 0.48 | 0.83 | **<0.01** | 0.85 |
|  | Leaf nitrogen/phosphorus ratio | 593 | <0.01 | 74.81 | 0.76 | 0.99 | **<0.01** | 0.62 |
|  | Leaf mean width | 593 | 5.50 | 428.7 | 1.25 | **0.02** | **<0.01** | 0.27 |
|  | Leaf phosphorus content | 593 | 0.81 | 48.65 | 0.80 | 0.37 | **<0.01** | 0.59 |

**FIGURE S1** Design of our common garden experiment. Shown are (a) assemblage types at different plant diversity levels and b) experiment design. Numbers with different colors in panel (a) represent different plant combinations, numbers in the brackets represent species richness, and numbers with different colours in panel (a) represent different species. SR, species richness. Fast/Slow species were classified by the specific-species functional traits based on the results of principal component analysis (Fig. S4).


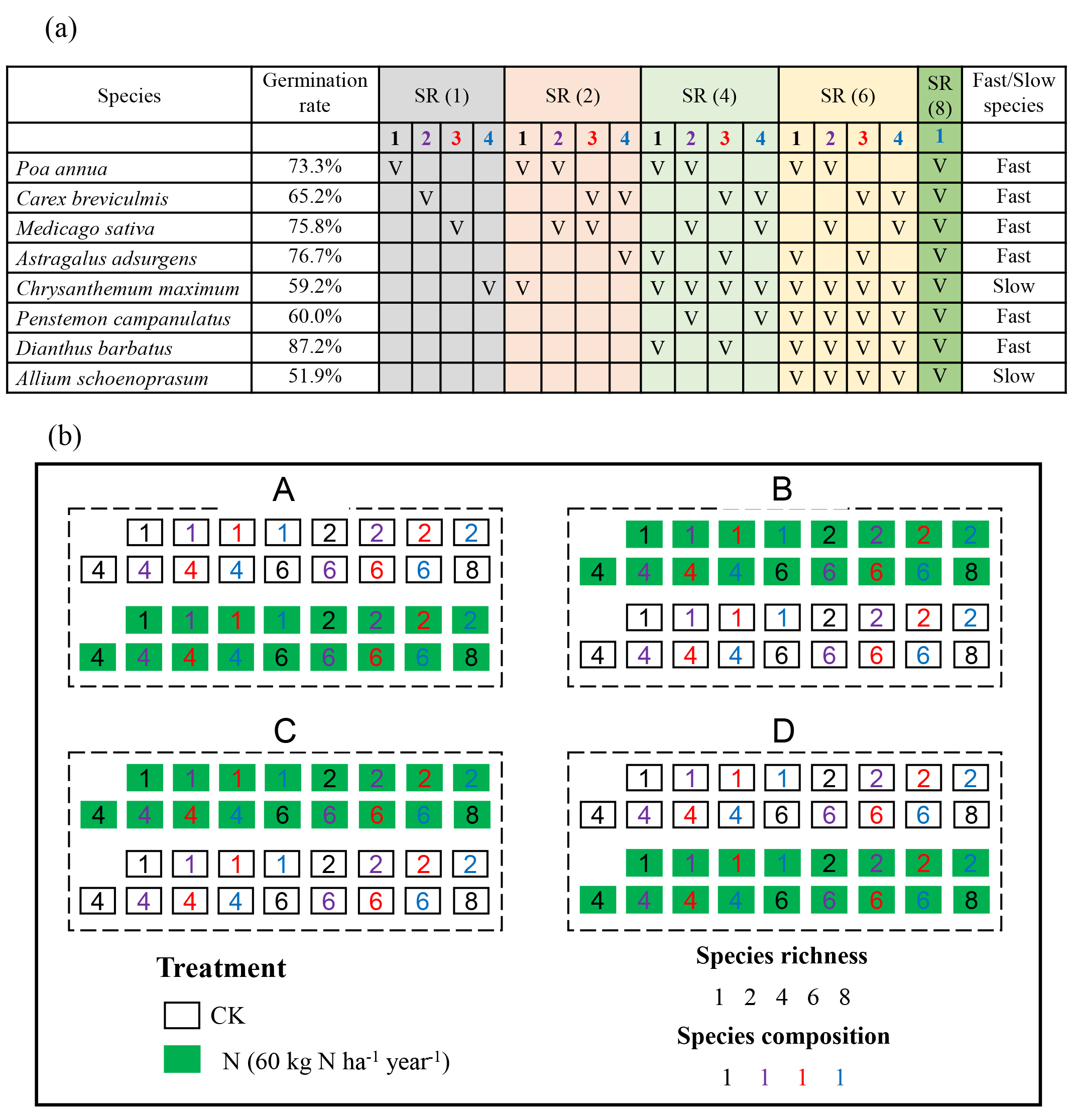


**FIGURE S2** Relationships between real diversity in plots and initial diversity. The points and lines with different colors represent different experimental years. The shading areas indicate the 95% confidence interval for the regression lines.


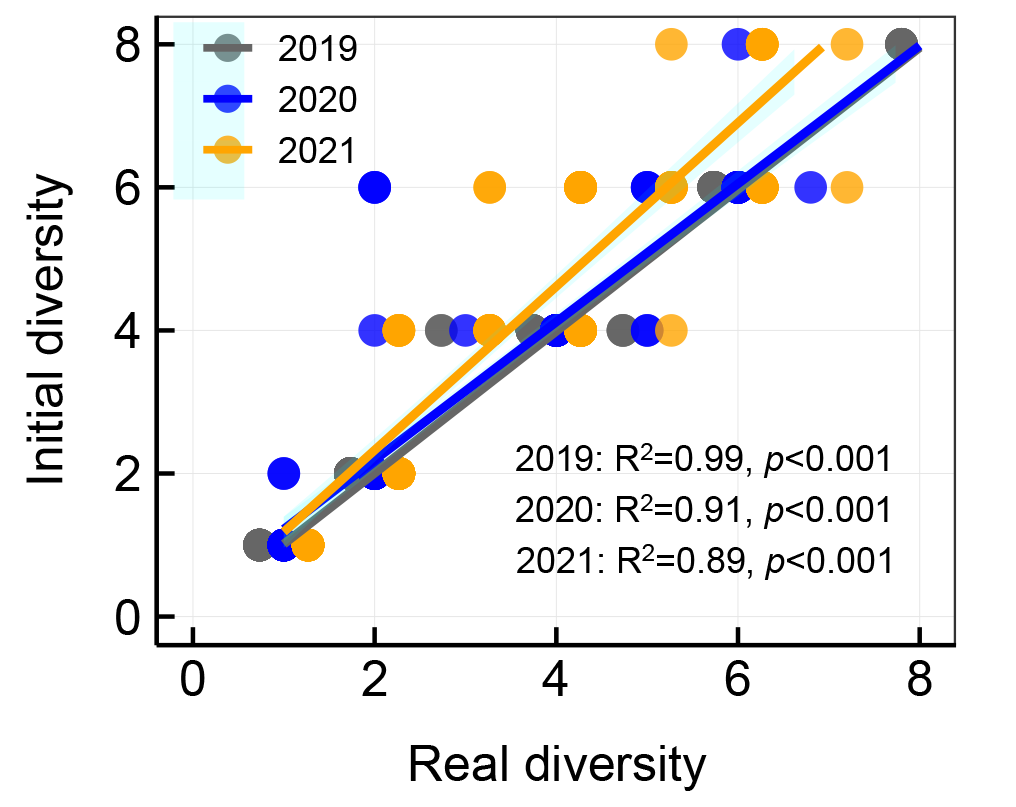


**FIGURE S3** Results of the principal component analysis for 12 community weighted mean (CWM) of plant traits. Black dots represent the 136 plots, and blue arrows depict the CWM. Variance explained by each principal component is shown in brackets. Points with different colours represent different treatments. LW: leaf mean width; LA: leaf area; LDMC: leaf dry matter content; SLA: specific leaf area; LNC: leaf nitrogen content; LPC: leaf phosphorus content; N/P: leaf nitrogen/phosphorus ratio; height, plant height.


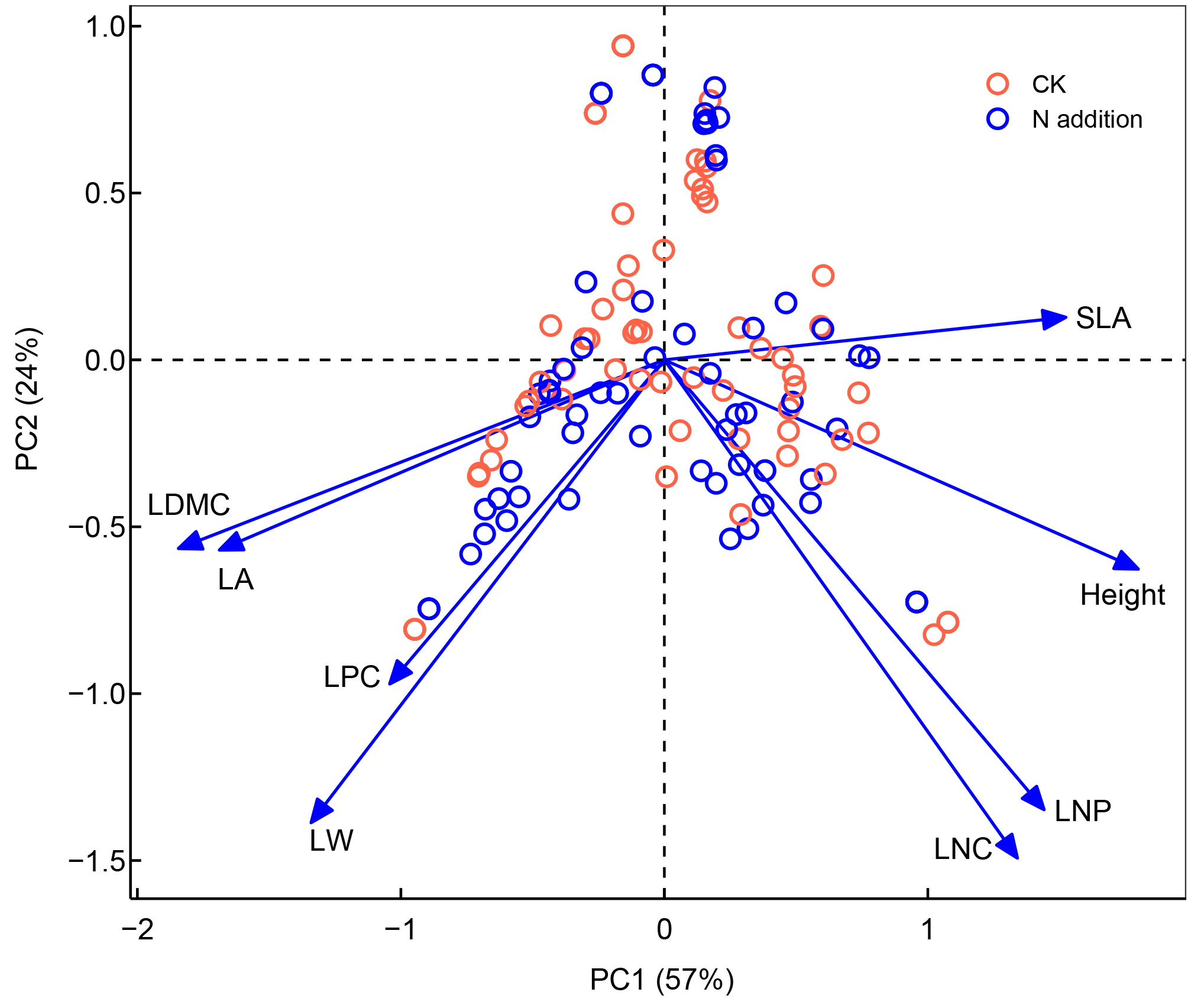


**FIGURE S4** Results of the principal component analysis for 12 functional trait of eight plant species. Variance explained by each principal component is shown in brackets. Points with different shapes represent different species. LW: leaf mean width; LA: leaf area; LDMC: leaf dry matter content; SLA: specific leaf area; LNC: leaf nitrogen content; N/P: leaf nitrogen/phosphorus ratio; height, plant height. AA, *Astragalus adsurgens*; AS, *Allium schoenoprasum*; CB, *Carex breviculmis*; CM, *Chrysanthemum maximum*; DB, *Dianthus barbatus*; MS, *Medicago sativa*; PA, *Poa annua*; PC, *Penstemon campanulatus*.


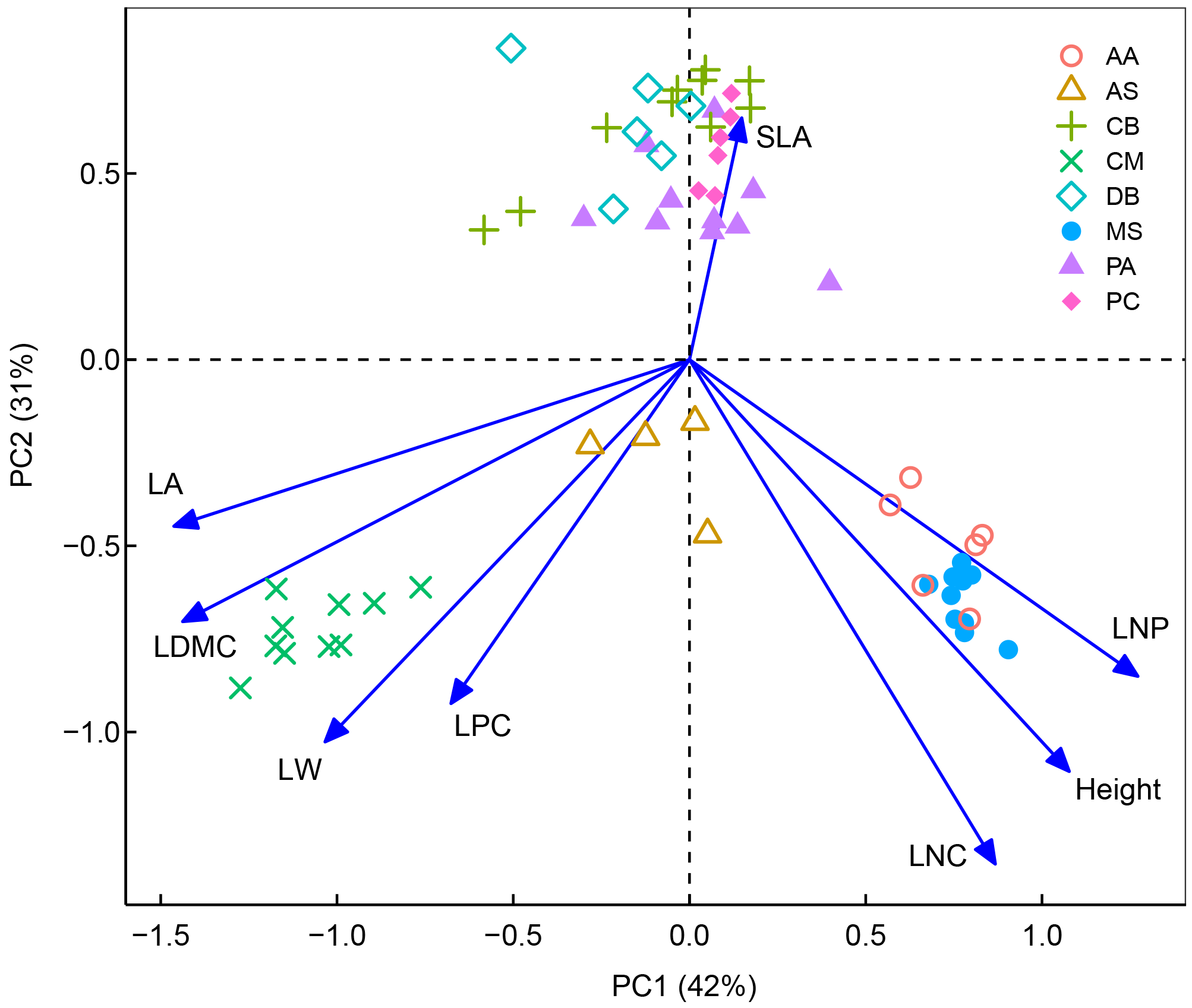


**FIGURE S5** Temporal mean productivity in relation to fast-slow functional diversity (Fast-slow FD, a) and community-weighted mean of fast-slow traits (CWM Fast-Slow, c); standard deviation of productivity in relation to fast-slow functional diversity (Fast-slow FD, b) and community-weighted mean of fast-slow traits (CWM Fast-Slow, d). The points with different colors and shapes represent different nitrogen addition levels. Solid and dashed lines represent significant (*p* ≤ 0.05) and insignificant (*p* > 0.05) relationships, respectively. The shading areas indicate 95% confidence interval for the regression lines. CK, control; N, nitrogen.


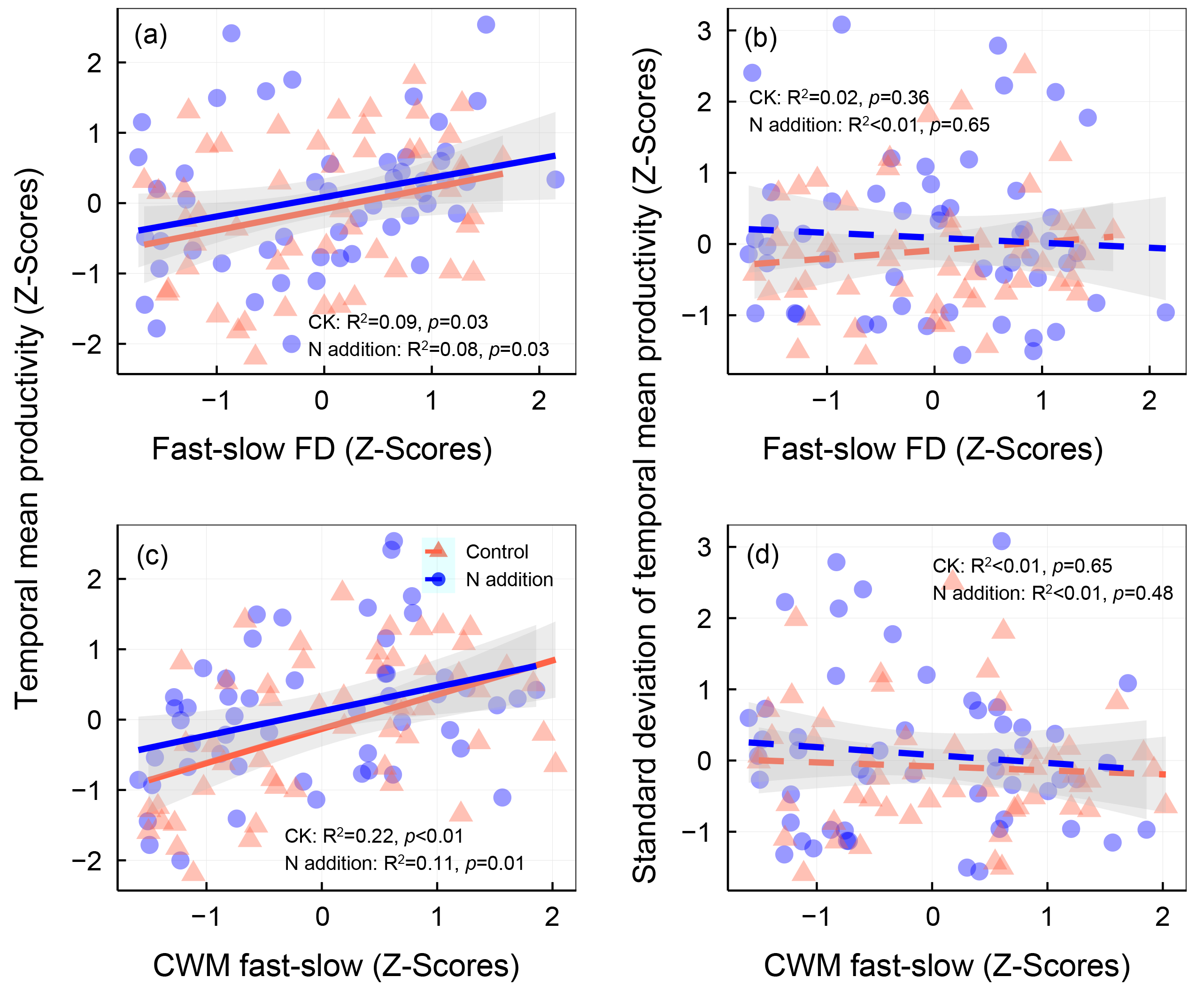


**FIGURE S6** Temporal mean productivity in relation to community weighted mean (CWM) of plant height (a), leaf area (b), leaf dry matter content (c), specific leaf area (d), leaf nitrogen content (e), and leaf nitrogen/phosphorus ratio (f). The points with different colors and shapes represent different nitrogen addition levels. Solid and dashed lines represent significant (*p* ≤ 0.05) and insignificant (*p* > 0.05) relationships, respectively. The shading areas indicate 95% confidence interval for the regression lines. CK, control; N, nitrogen.


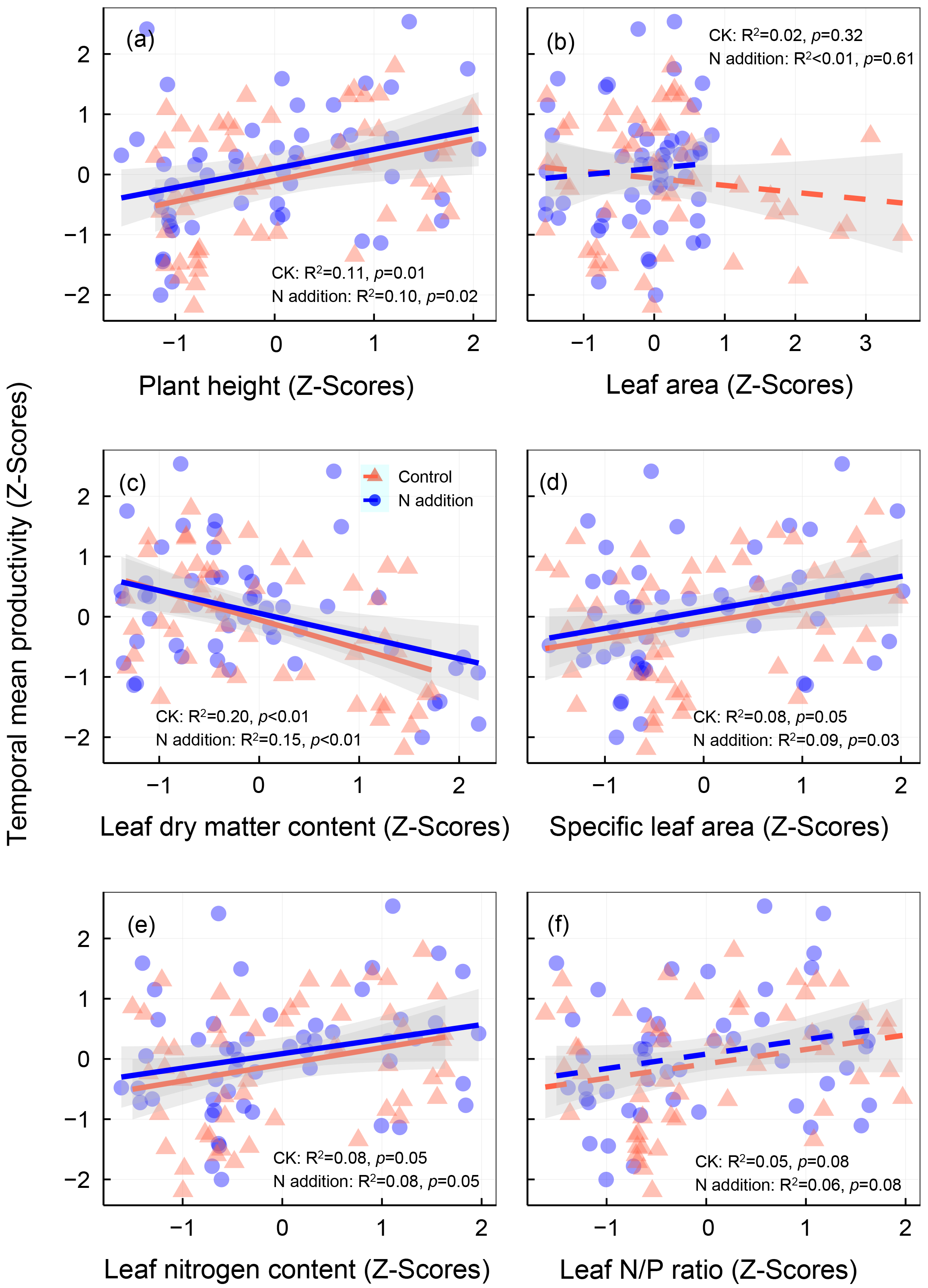


**FIGURE S7** Standard deviation of temporal mean productivity in relation to community-weighted mean (CWM) of plant height (a), leaf area (b), leaf dry matter content (c), specific leaf area (d), leaf nitrogen content (e), and leaf nitrogen/phosphorus ratio (f). The points with different colors and shapes represent different nitrogen addition levels. Solid and dashed lines represent significant (*p* ≤ 0.05) and insignificant (*p* > 0.05) relationships. The shading areas indicate 95% confidence interval for the regression lines. CK, control; N, nitrogen.


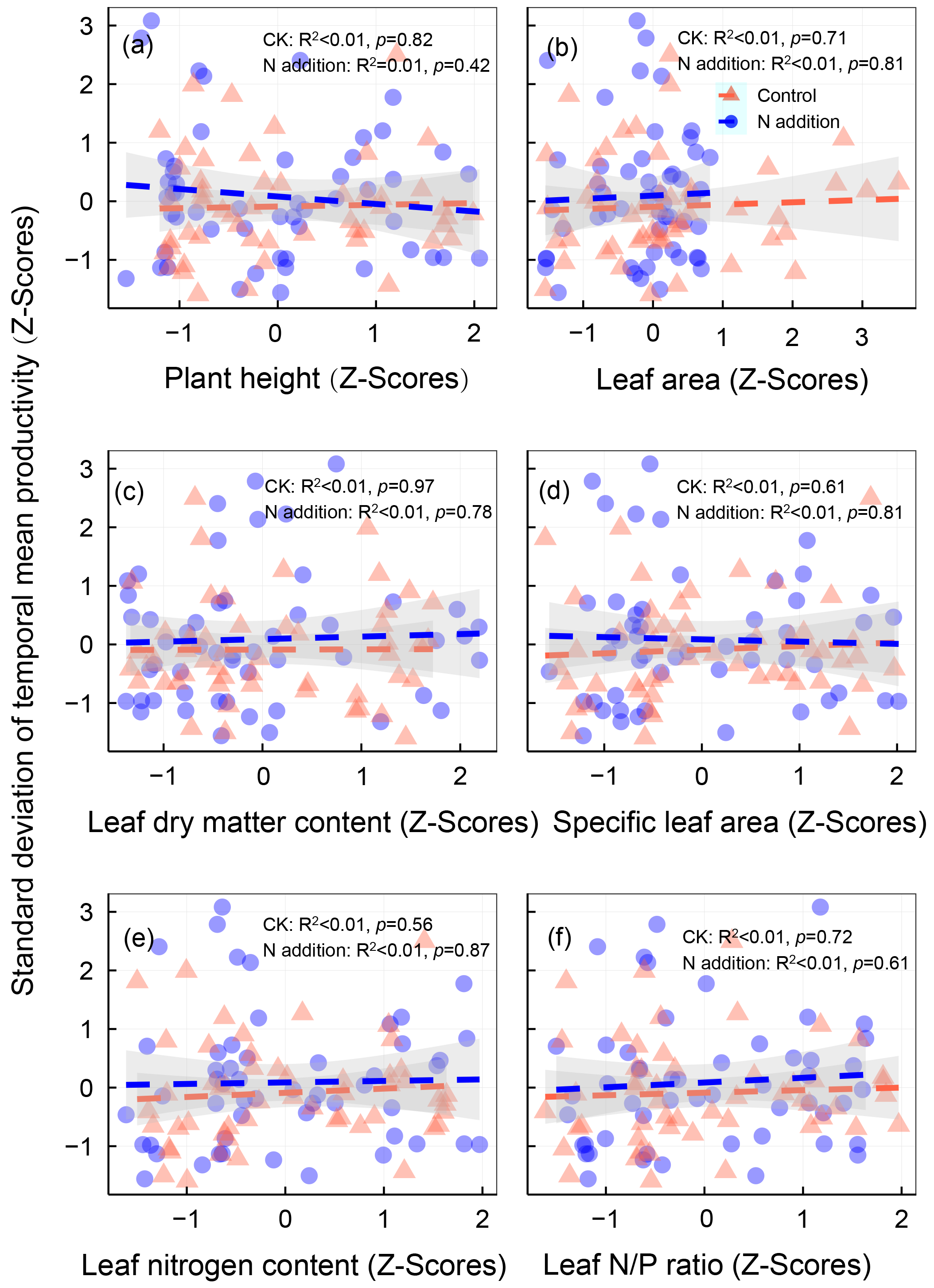


**FIGURE S8** The relationships between population stability and fast-slow trait of different species. The points with different colors represent different species. Solid and dashed lines represent significant (*p* ≤ 0.05) and insignificant (*p* > 0.05) relationships. The shading areas indicate 95% confidence interval for the regression lines. AA, *Astragalus adsurgens*; AS, *Allium schoenoprasum*; CA, *Carex breviculmis*; CM, *Chrysanthemum maximum*; DB, *Dianthus barbatus*; MS, *Medicago sativa*; PA, *Poa annua*; PC, *Penstemon campanulatus*.


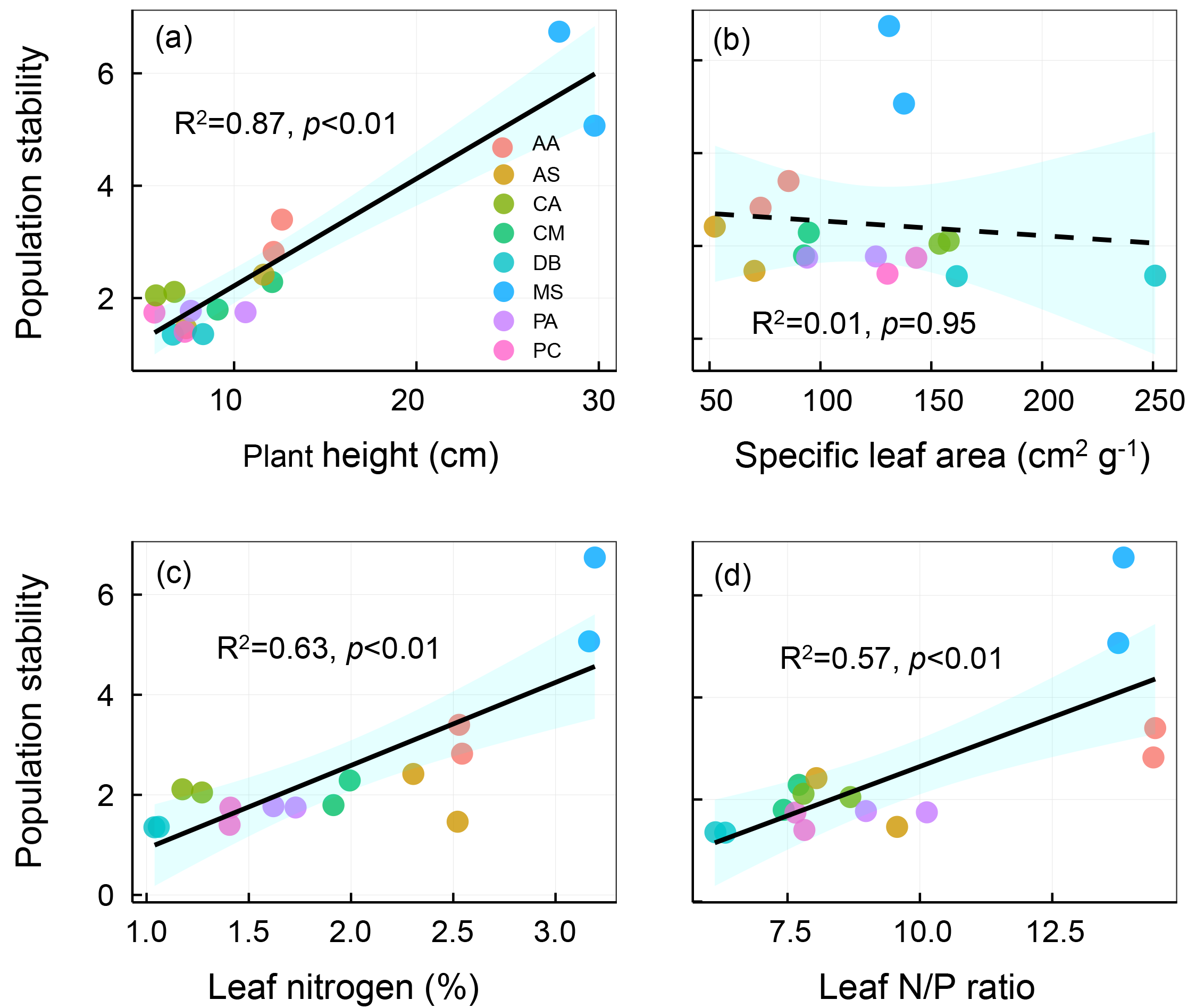


**FIGURE S9** Multi-groups structural equation model exploring the effects of plant species richness, fast–slow functional diversity (fast–slow FD), and community-weighted mean of plant height (a), leaf area (b), leaf dry matter content (LDMC, c), specific leaf area (SLA, d), leaf nitrogen content (e) and nitrogen/phosphorus ratio (f) on species asynchrony, mean and variation (s.d. of productivity) in productivity and community stability in the control and nitrogen addition treatments. Boxes represent measured variables and arrows represent relationships among variables. Solid and dashed arrows represent positive and negative pathways. Black, red, and blue arrows represent significant (*p* ≤ 0.05) pathways in the overall, control, and nitrogen addition treatment, respectively. Grey arrows represent insignificant pathways (*p* > 0.05). Standardized path coefficients are given next to each path.


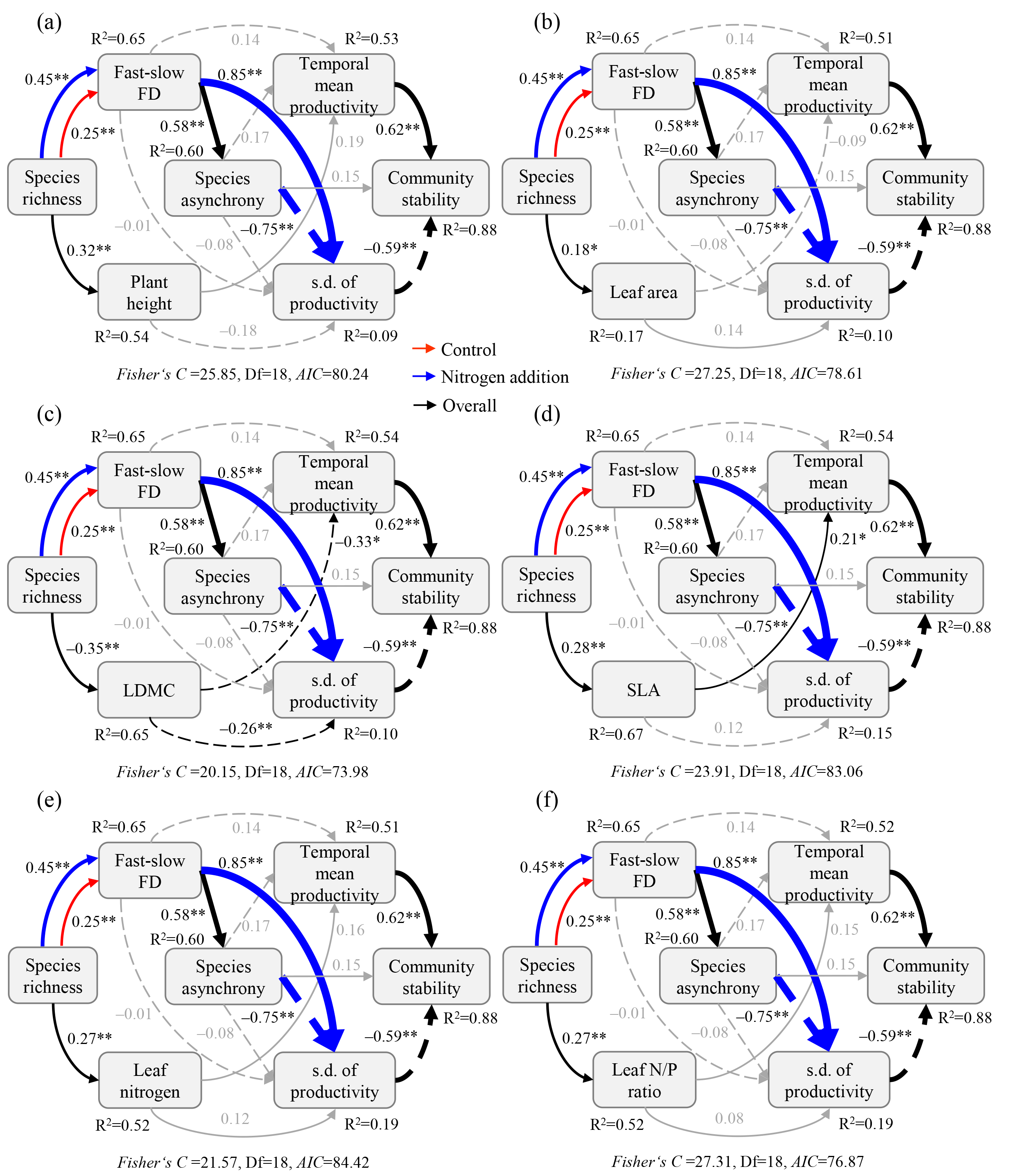


**FIGURE S10** Effects of N addition on fast-slow trait of different species. The bars with different colors represent different nitrogen addition levels. Different letters represent the difference between the control and nitrogen addition treatments. AA, *Astragalus adsurgens*; AS, *Allium schoenoprasum*; CB, *Carex breviculmis*; CM, *Chrysanthemum maximum*; DB, *Dianthus barbatus*; MS, *Medicago sativa*; PA, *Poa annua*; PC, *Penstemon campanulatus*; SLA, specific leaf area; N, nitrogen; P, phosphorus.


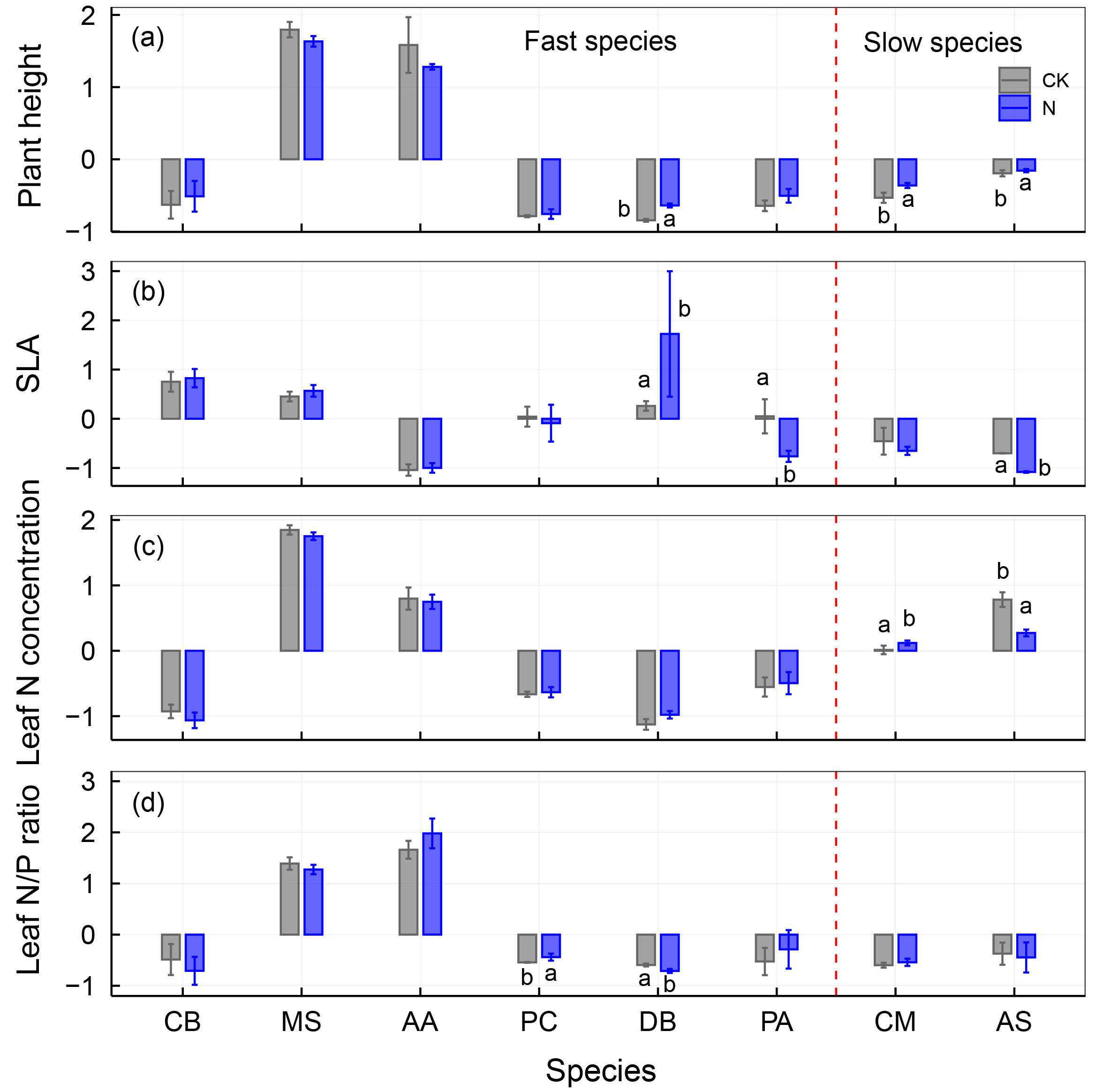

Supplement: Supplementary file 1 [file DataSheet_1.docx]
